# Development and Validation of Nomograms to Predict Cancer-Specific Survival and Overall Survival in Elderly Patients With Prostate Cancer: A Population-Based Study

**DOI:** 10.3389/fonc.2022.918780

**Published:** 2022-06-23

**Authors:** Zhaoxia Zhang, Chenghao Zhanghuang, Jinkui Wang, Xiaomao Tian, Xin Wu, Maoxian Li, Tao Mi, Jiayan Liu, Liming Jin, Mujie Li, Dawei He

**Affiliations:** ^1^ Department of Urology, Chongqing Key Laboratory of Children Urogenital Development and Tissue Engineering, Chongqing, China; ^2^ Chongqing Key Laboratory of Pediatrics, Ministry of Education Key Laboratory of Child Development and Disorders, Chongqing, China; ^3^ National Clinical Research Center for Child Health and Disorders, China International Science and Technology Cooperation base of Child development and Critical Disorders; Children’s Hospital of Chongqing Medical University, Chongqing, China; ^4^ Department of Urology, Kunming Children’s Hospital, Yunnan Provincial Key Research Laboratory of Pediatric Major Diseases, Kunming, China

**Keywords:** nomogram, old age, prostate cancer, CSS, SEER

## Abstract

**Objective:**

Prostate cancer (PC) is the most common non-cutaneous malignancy in men worldwide. Accurate predicting the survival of elderly PC patients can help reduce mortality in patients. We aimed to construct nomograms to predict cancer-specific survival (CSS) and overall survival (OS) in elderly PC patients.

**Methods:**

Information on PC patients aged 65 years and older was downloaded from the Surveillance, Epidemiology, and End Results (SEER) database. Univariate and multivariate Cox regression models were used to determine independent risk factors for PC patients. Nomograms were developed to predict the CSS and OS of elderly PC patients based on a multivariate Cox regression model. The accuracy and discrimination of the prediction model were tested by the consistency index (C-index), the area under the subject operating characteristic curve (AUC), and the calibration curve. Decision curve analysis (DCA) was used to test the clinical value of the nomograms compared with the TNM staging system and D’Amico risk stratification system.

**Results:**

135183 elderly PC patients in 2010-2018 were included. All patients were randomly assigned to the training set (N=94764) and the validation set (N=40419). Univariate and multivariate Cox regression model analysis revealed that age, race, marriage, histological grade, TNM stage, surgery, chemotherapy, radiotherapy, biopsy Gleason score (GS), and prostate-specific antigen (PSA) were independent risk factors for predicting CSS and OS in elderly patients with PC. The C-index of the training set and the validation set for predicting CSS was 0.883(95%CI:0.877-0.889) and 0.887(95%CI:0.877-0.897), respectively. The C-index of the training set and the validation set for predicting OS was 0.77(95%CI:0.766-0.774)and 0.767(95%CI:0.759-0.775), respectively. It showed that the proposed model has excellent discriminative ability. The AUC and the calibration curves also showed good accuracy and discriminability. The DCA showed that the nomograms for CSS and OS have good clinical potential value.

**Conclusions:**

We developed new nomograms to predict CSS and OS in elderly PC patients. The models have been internally validated with good accuracy and reliability and can help doctors and patients to make better clinical decisions.

## Background

Prostate cancer (PC) is the most common non-dermatological tumor in men worldwide. In 2022, the number of new prostate cancer patients in the United States will reach 268,490 (1). Most prostate cancer can be diagnosed early due to the popularity of prostate-specific antigen (PSA) screening and biopsy testing techniques. The treatment of prostate cancer mainly includes radical prostatectomy, androgen deprivation therapy (ADT), radiotherapy, and chemotherapy, which dramatically improves prostate cancer patients’ survival rate. At the same time, the total number of prostate cancer patients is also increasing. As of 2020, there were 3.65 million confirmed PC patients diagnosed in the United States, and the number is expected to increase to 5.02 million by 2030 (2). It should be noted that mortality rates among prostate cancer patients also rank second among cancer deaths worldwide due to their high morbidity. Although most patients with PC have a good prognosis, some patients still have a recurrence and distant metastasis, making significant differences in the prognosis of PC. In 2022,34,500 people are expected to die from PC in the United States or about 11% of male cancer deaths (1).

Previously, the US Joint Commission on Cancer (AJCC) tumor-lymph node-metastatic (TNM) cancer staging system was used for the effective management of a variety of cancers (3). However, a growing number of studies have shown that the TNM stage alone does not accurately predict patient outcomes because of multiple factors clinically associated with PC prognosis (4–6), especially the Gleason score(GS) and prostate-specific antigen (PSA). GS is the most powerful tool for predicting outcomes of PC (7), developed by Donald Gleason Joint Urology Research Group between 1966 and 1974 (8) and revised in 2005 and 2014 (9, 10). PSA is mainly used for the screening of PC, causing a significant increase in the detection rate of PC. It is well known that PSA level is an essential factor in determining the aggressiveness of prostate cancer (11), and some studies show that PSA level is considered an essential prognostic factor in PC, with a linear relationship between PSA and PC prognosis (12, 13). However, some studies have shown that PSA screening does not reduce all-cause mortality in patients with PC (14, 15). Although the combination of PSA, histological grade, and TNM staging system can establish prognostic models, refining the stratification system can improve the discriminatory ability of prognostic models (16, 17). However, this model still cannot evaluate the impact of critical clinical variables, including age, marriage, race, and treatment mode, on the prognosis of PC patients.

A nomogram is a digital graphical tool that can predict the occurrence probability of a given event based on the data of known variables. It is considered superior to the conventional TNM staging system (18, 19). It has been widely used to predict the prognosis of multiple cancers, including glioma, bladder cancer, renal carcinoma, mammary cancer, and Colon cancer (20–24). There are also some nomograms for PC, but primarily for distant metastatic PC, PC with particular bone metastases, or patients with nonmetastatic PC. There are also nomograms designed specifically for PC patients with GS 3 + 4 and 4 + 3 scores (25–28).Elderly patients are a group with high incidence and mortality from PC, with a median age at diagnosis of 66 years (29). Moreover, with the aging population, the base of the elderly is also expanding, and the cancer health management of the elderly has become a major problem that cannot be ignored (30). More than 60 percent of PC patients are over the age of 65, and more than 90 percent of PC deaths occur in this age group (6), but there is no nomogram for PC patients more than 65 years old. Consider that elderly PC patients lead to many non-cancer-specific deaths due to comorbidities, affecting overall survival (OS). Therefore, our study aims to identify independent prognostic factors for elderly PC patients using the Surveillance, Epidemiological, and End Results (SEER) database and develop and validate nomograms for specific survival (CSS) and OS in elderly PC patients, as well as to provide a reference basis for the clinical diagnosis and treatment work.

## Patients and Methods

### Data Source and Data Extraction

We downloaded patient data from the SEER database, including patients aged 65 years and older diagnosed with PC between 2010 and 2018. The SEER database is a national cancer database containing 18 cancer registries covering approximately 30% of the population. Since the patient information in the SEER database is anonymized and the data is publicly available, ethical approval and patient informed consent were not required for our study. The research methodology used in this study follows the research guidelines published in the SEER database.

**Figure 1:**
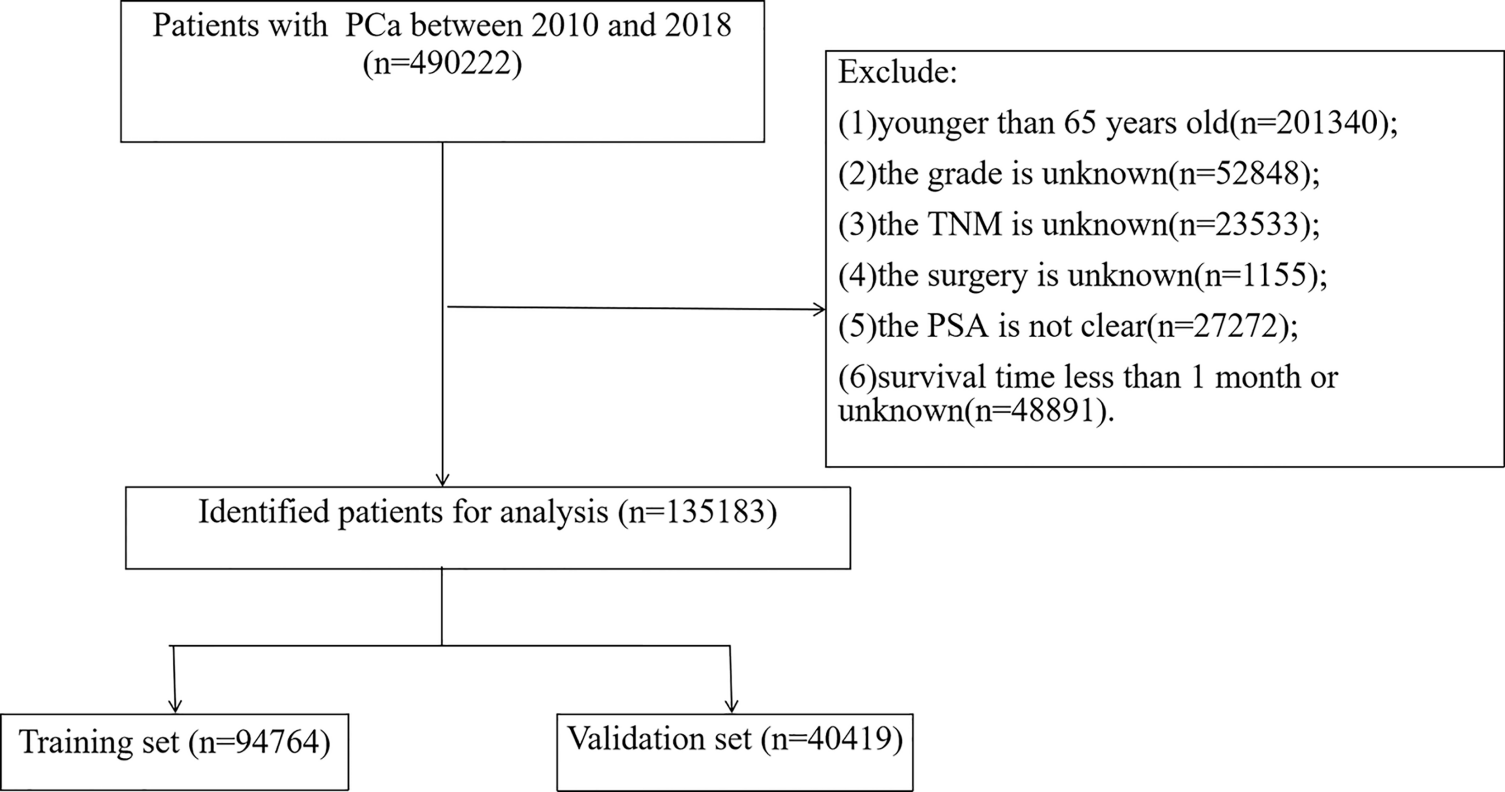
Flowchart for inclusion and exclusion of elderly patients with PC.

We collected clinicopathological information for all elderly PC patients, including age, race, year of diagnosis, marital status, histological tumor grade, TNM stage, surgery, radiotherapy, chemotherapy, PSA, and biopsy GS. Patient follow-up results, including survival status, cause of death, and survival time, are also available from the SEER database. Inclusion criteria: (1) patients age≥ 65;(2) with a pathological diagnosis of PC. Exclusion criteria: (1) patients younger than 65 years old; (2) tumor grade is unknown; (3) TNM stage is unknown; (4) surgical method is unknown; (5) PSA is not clear; (6) survival time is less than one month or survival time is unknown. The flowchart of patient inclusion and exclusion is shown in **Figure 1**.

Patients were classified as white, Black, and other types (American Indian/AK Native, Asian/Pacific Islander). The histological classification of tumors includes high differentiation (grade I), moderate differentiation (grade II), low differentiation (grade III), and undifferentiated (grade IV). According to the SEER surgical code, the surgical methods are divided into non-surgical surgery (surgical code 0), local tumor resection (surgical code 10-30), and radical prostatectomy (surgical code 50-80).

### Development and Validation of the Nomograms

We first randomly divided the patients into two groups for the development and internal validation of the nomogram. All data were randomly divided into the training set (70%) and the validation set (30%). In addition, the data of PC patients from 2016-to 2018 were externally validated in time. Univariate and multivariate Cox proportional regression models were used to identify independent risk factors affecting patients’ outcomes in the training set. We constructed nomograms based on a multivariate Cox regression model for predicting CSS and OS at 3-,5-, and 8-year. The calibration curves for 1,000 bootstrap samples were used to verify the accuracy of the nomograms. The consistency index (c-index) and the area under the subject operating characteristic curve (AUC) were used to test the accuracy and discrimination of the models.

### Clinical Application

We use the decision analysis curve (DCA) to evaluate the clinical value of the nomograms for predicting CSS and OS at 3-,5-, and 8- years compared with the TNM staging system and D’Amico risk stratification system. Furthermore, we also calculated the risk for each patient from the nomograms. All patients were divided into high-risk and low-risk groups based on the cutoff value of the subject operating characteristic curve (ROC). The production principle of the ROC curve is to set several different critical values for the variable, calculate the corresponding sensitivity (sensitivity) and specificity (specificity) at each critical value, and then take the sensitivity as the ordinate and 1-specificity as the abscissa to draw the curve. Our ROC curves are time-dependent and are time-dependent. We used time-based ROC curves to test the nonlinear relationship of CSS or OS with risk values in the dataset and sought the closest risk score to HR=1 on the ROC curve as the cutoff. The Log-rank test and Kaplan-Meier (K-M) curves examined the differences in survival between high-risk and low-risk patients. In addition, surgical differences among patients in different risk groups were also analyzed.

### Statistical Analysis

Continuous variables(age) were tested for normal distribution and conform to the normal distribution, described by the mean ± standard deviation. Chi-square or non-parametric U tests were used for comparison between groups. Other categorical variables were described by frequency (%), and the groups were compared using the chi-square test. The Cox regression models analyzed patient prognostic factors, and the log-rank test and K-M curves analyzed the survival differences of patients. All statistical methods were performed using R software version 4.1.0 and SPSS26.0. The R packages including “DynNom”, “RMS”, “Survival”, and “ggDCA” were used. A P value less than 0.05 was considered statistically significant.

## Result

### Clinical Features

A total of 135,183 patients between 2010 and 2018 were included in this study. All patients were randomly assigned to the training set (N=94764) and the validation set (N=40419). The mean age of both groups was 71.6 ± 5.1 years, and most of both groups were white (79.4%) and married (67.8%). The tumors included grade I (14.8%), II (40.3%), III (43.5%), and IV (1.44%).Patients with stage T1 (45.7%), T2 (39.1%), T3 (13.4%), and T4 (1.73%).Most patients were in stage N0 (95.8%) and staged M0 (95.5%).Patients with Non-surgical treatment (65.0%), patients who underwent local tumor resection (5.93%), and patients who underwent radical prostatectomy (29.1%).99% of patients received chemotherapy. 39.8% received radiotherapy, while 60.2% did not. Most patients had an unknown biopsy GS score (71.1%), and biopsy GS7 was about 20%. PSA 4-10ng/ml(57.1%), >10ng/ml(33.2%). The data did not show significant statistical bias in both groups, and the results are shown in **Table 1**.

**Table 1:** Clinicopathological characteristics of elderly patients with PCa.

	All	Training cohort	Validation cohort	p
	N = 135183	N = 94764	N = 40419	
Age	71.6 (5.51)	71.5 (5.48)	71.6 (5.53)	0.159
Race:				0.483
white	107381 (79.4%)	32025 (79.2%)	75356 (79.5%)	
black	16005 (11.8%)	4826 (11.9%)	11179 (11.8%)	
other	11797 (8.73%)	3568 (8.83%)	8229 (8.68%)	
Marital:				0.331
No	43594 (32.2%)	13111 (32.4%)	30482 (32.2%)	
Married	91590 (67.8%)	27308 (67.6%)	64282 (67.8%)	
Grade:				0.252
I	20021 (14.8%)	5880 (14.5%)	14141 (14.9%)	
II	54426 (40.3%)	16308 (40.3%)	38118 (40.2%)	
III	58791 (43.5%)	17666 (43.7%)	41125 (43.4%)	
IV	1945 (1.44%)	565 (1.40%)	1380 (1.46%)	
T:				0.516
T1	61819 (45.7%)	18482 (45.7%)	43337 (45.7%)	
T2	52878 (39.1%)	15889 (39.3%)	36989 (39.0%)	
T3	18142 (13.4%)	5368 (13.3%)	12774 (13.5%)	
T4	2344 (1.73%)	680 (1.68%)	1664 (1.76%)	
N:				0.603
N0	129460 (95.8%)	38726 (95.8%)	90734 (95.7%)	
N1	5723 (4.23%)	1693 (4.19%)	4030 (4.25%)	
M:				0.501
M0	129046 (95.5%)	38560 (95.4%)	90486 (95.5%)	
M1	6137 (4.54%)	1859 (4.60%)	4278 (4.51%)	
Surgery:				0.351
No	87843 (65.0%)	26354 (65.2%)	61489 (64.9%)	
Local tumor excision	8019 (5.93%)	2348 (5.81%)	5671 (5.98%)	
Radical prostatectomy	39321 (29.1%)	11717 (29.0%)	27604 (29.1%)	
Chemotherapy:				0.885
No	133822 (99.0%)	40015 (99.0%)	93807 (99.0%)	
Yes	1361 (1.01%)	404 (1.00%)	957 (1.01%)	
Radiation:				0.530
No	81407 (60.2%)	24288 (60.1%)	57119 (60.3%)	
Yes	53776 (39.8%)	16131 (39.9%)	37645 (39.7%)	
Gleason:				0.486
≤6	5141 (3.80%)	1549 (3.83%)	3592 (3.79%)	
3+4	17182 (12.7%)	5185 (12.8%)	11997 (12.7%)	
4+3	9542 (7.06%)	2819 (6.97%)	6723 (7.09%)	
≥8	7217 (5.34%)	2102 (5.20%)	5115 (5.40%)	
Unknown	96101 (71.1%)	28764 (71.2%)	67337 (71.1%)	
PSA:				0.245
<4	13169 (9.74%)	3966 (9.81%)	9203 (9.71%)	
4-10	77140 (57.1%)	23168 (57.3%)	53972 (57.0%)	
>10	44874 (33.2%)	13285 (32.9%)	31589 (33.3%)	
CSS:				0.680
Dead	6184 (4.57%)	1864 (4.61%)	4320 (4.56%)	
Alive	128999 (95.4%)	38555 (95.4%)	90444 (95.4%)	
Survival.months	46.2 (29.9)	46.3 (29.9)	46.1 (29.9)	0.146

### Univariate and Multivariate COX Regression Analysis

Univariate Cox regression models were first used in training set to analyze and screen for factors associated with patient survival. The results showed that these factors, including age, race, marriage, tumor grade, TNM stage, surgery, chemotherapy, radiotherapy, PSA, and biopsy GS, could all affect patient survival. Then, multivariate Cox regression models were used to screen for independent risk factors associated with CSS and OS of elderly PC patients. The results showed that age, race, marriage, tumor grade, TNM stage, surgery, radiotherapy, chemotherapy, PSA, and biopsy GS were prognostic factors affecting patient CSS and OS. The analysis results are shown in **Tables 2** and **3**.

**Table 2:** Univariate and multivariate analyses of CSS in training cohort.

	Univariate			Multivariate		
	HR	95%CI	P	HR	95%CI	P
Age	1.12	1.12-1.13	<0.001	1.055	1.051-1.059	<0.001
Race						
white						
black	1.29	1.18-1.41	<0.001	1.174	1.089-1.264	<0.001
other	0.8	0.71-0.89	<0.001	0.662	0.599-0.732	<0.001
Marital						
No						
Married	0.66	0.63-0.71	<0.001	0.849	0.807-0.895	<0.001
Grade						
I						
II	1.87	1.48-2.36	<0.001	1.521	1.262-1.835	<0.001
III	9.78	7.85-12.18	<0.001	3.963	3.307-4.749	<0.001
IV	20.3	13.63-30.22	<0.001	6.414	4.58-8.983	<0.001
T						
T1						
T2	1.13	1.05-1.21	0.001	1.196	1.127-1.27	<0.001
T3	1.43	1.3-1.57	<0.001	1.389	1.271-1.518	<0.001
T4	11.37	10.3-12.55	<0.001	2.216	2.019-2.432	<0.001
N						
N0						
N1	8.13	7.54-8.77	<0.001	1.365	1.269-1.468	<0.001
M						
M0						
M1	27.62	25.96-29.4	<0.001	7.254	6.773-7.769	<0.001
Surgery						
No						
Local tumor excision	2.25	2.06-2.45	<0.001	1.609	1.494-1.732	<0.001
Radical prostatectomy	0.24	0.21-0.27	<0.001	0.759	0.541-1.064	0.109
Chemotherapy						
No						
Yes	10.64	9.42-12.02	<0.001	1.41	1.268-1.567	<0.001
Radiation						
No						
Yes	0.64	0.6-0.68	<0.001	0.647	0.609-0.688	<0.001
PSA						
<4						
4-10	0.71	0.62-0.81	<0.001	0.798	0.711-0.895	<0.001
>10	4.16	3.66-4.73	<0.001	1.559	1.396-1.741	<0.001
Gleason						
≤6						
3+4	0.88	0.55-1.41	0.592	0.511	0.348-0.748	0.001
4+3	1.35	0.83-2.2	0.231	0.608	0.405-0.913	0.016
≥8	8.18	5.4-12.4	<0.001	2.419	1.711-3.422	<0.001
Unknown	10.43	7.04-15.45	<0.001	2.699	1.712-4.253	<0.001

**Table 3:** Univariate and multivariate analyses of OS in training cohort.

	Univariate			Multivariate		
	HR	95%CI	P	HR	95%CI	P
Age	1.12	1.11-1.12	<0.001	1.073	1.07-1.075	<0.001
Race						
white						
black	1.29	1.22-1.35	<0.001	1.245	1.192-1.301	<0.001
other	0.69	0.65-0.75	<0.001	0.632	0.595-0.672	<0.001
Marital						
No						
Married	0.66	0.64-0.68	<0.001	0.798	0.774-0.822	<0.001
Grade						
I						
II	1.24	1.14-1.36	<0.001	1.158	1.078-1.244	<0.001
III	2.57	2.37-2.79	<0.001	1.767	1.645-1.898	<0.001
IV	3.78	2.84-5.03	<0.001	2.487	1.966-3.146	<0.001
T						
T1						
T2	0.96	0.92-1	0.04	1.127	1.089-1.166	<0.001
T3	0.86	0.81-0.92	<0.001	1.217	1.147-1.292	<0.001
T4	4.41	4.07-4.77	<0.001	1.889	1.754-2.034	<0.001
N						
N0						
N1	3.51	3.31-3.74	<0.001	1.261	1.188-1.338	<0.001
M						
M0						
M1	8.82	8.42-9.24	<0.001	3.241	3.087-3.403	<0.001
Surgery						
No						
Local tumor excision	1.89	1.79-1.99	<0.001	1.373	1.308-1.44	<0.001
Radical prostatectomy	0.33	0.31-0.35	<0.001	0.635	0.519-0.776	<0.001
Chemotherapy						
No						
Yes	4.7	4.21-5.23	<0.001	1.393	1.269-1.529	<0.001
Radiation						
No						
Yes	0.85	0.82-0.88	<0.001	0.716	0.692-0.741	<0.001
PSA						
<4						
4-10	0.8	0.75-0.85	<0.001	0.884	0.837-0.935	<0.001
>10	2.25	2.11-2.4	<0.001	1.283	1.213-1.358	<0.001
Gleason						
≤6						
3+4	0.7	0.6-0.81	<0.001	0.582	0.512-0.662	<0.001
4+3	0.72	0.61-0.86	<0.001	0.597	0.515-0.691	<0.001
≥8	1.58	1.35-1.84	<0.001	0.918	0.799-1.055	0.228
Unknown	2.91	2.58-3.29	<0.001	1.155	0.93-1.435	0.192

### Nomograms Development for the 3-Year, 5-Year, and 8-Year CSS and OS

**Figure 2:**
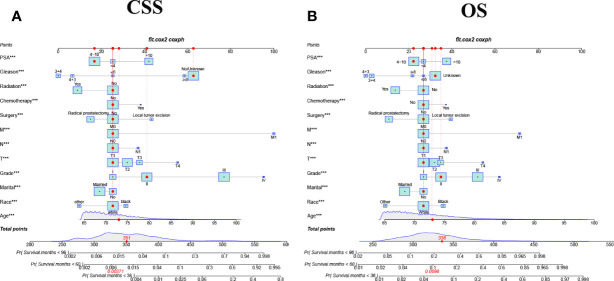
The nomograms for predicting 3-,5-,8-year CSS and OS in elderly patients with PC. **(A)** The nomogram for predicting CSS in elderly patients with PC. **(B)** The nomogram for predicting OS in elderly patients with PC. ***P < 0.001 vs. reference.

We constructed nomograms that predicted CSS and OS at 3-year,5-year, and 8-year in elderly PC patients based on multivariate Cox regression models **(** **Figure 2** **).** From the figure, age, TNM stage, tumor grade, surgery, PSA, and biopsy GS were the most influential factors for predicting CSS and OS in elderly PC patients. In addition, radiotherapy and chemotherapy are also essential factors. However, marriage and race had little effect on patient survival.

### Validation of the Nomograms

**Figure 3:**
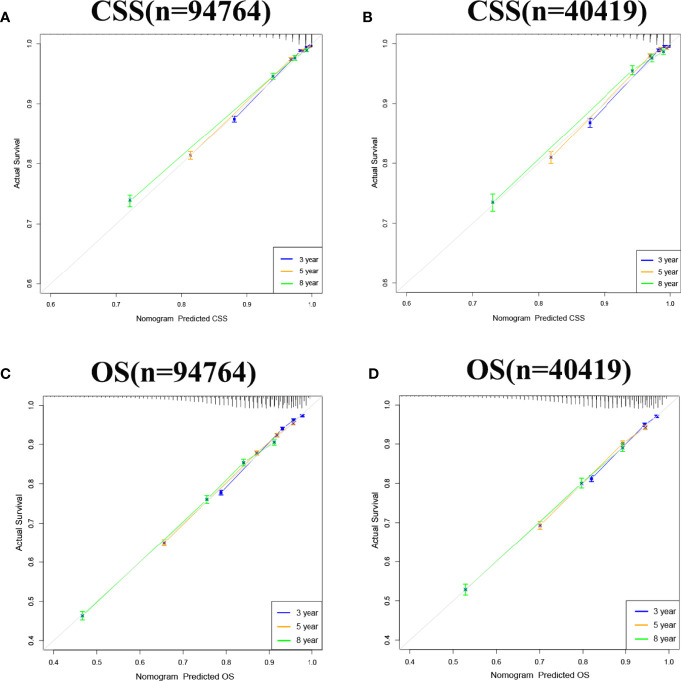
Calibration curve of the nomograms for predicting 3-,5-,8-year CSS and OS in elderly patients with PC. **(A)** Calibration curve of the nomograms for predicting 3-,5-,8-year CSS in the training set. **(B)** Calibration curve of the nomograms for predicting 3-,5-, and 8-year CSS in the validation set. **(C)** Calibration curve of the nomograms for predicting 3-,5-,8-year OS in the training set. **(D)** Calibration curve of the nomograms for predicting 3-,5-,8-year OS in the validation set. The horizontal axis is the predicted value in the nomogram, and the vertical axis is the observed value.

**Figure 4:**
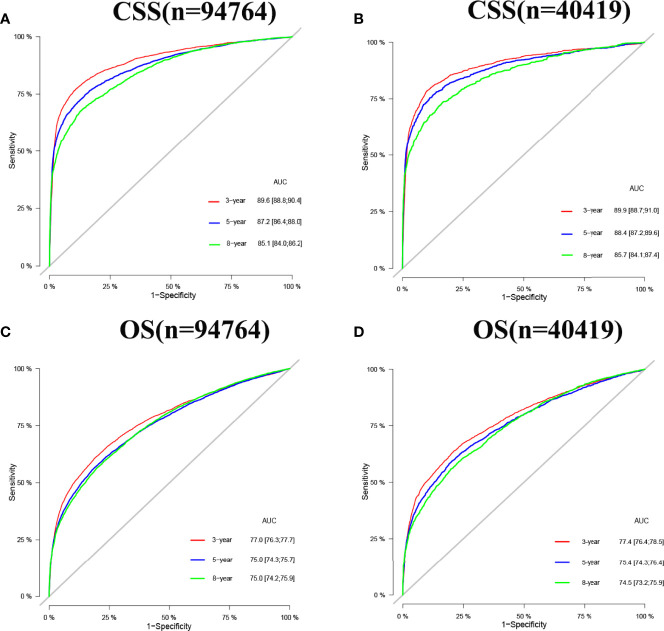
AUC for predicting 3-, 5-, and 8-year CSS and OS in elderly patients with PC. **(A)** The AUC at 3-, 5-, and 8-year for CSS in the training set was 89.6,87.2, and 85.1. **(B)** The AUC for CSS in the validation set was 89.9,88.4, and 85.7. **(C)** The AUC at 3-, 5- and 8-year for OS in the training set was 77.0,75.0, and 75.0. **(D)** The AUC at 3-, 5- and 8-year for OS in the validation set was 77.4,75.4, and 74.5.

Internal cross-validation was used to test the accuracy and discriminability of models. The C-index of the training set and the validation set for predicting CSS is 0.883(95%CI:0.877-0.889) and 0.887(95%CI:0.877-0.897), respectively. The C-index of the training set and the validation set for predicting OS was 0.77(95%CI:0.766-0.774)and 0.767(95%CI:0.759-0.775), respectively. It indicated that the nomograms for CSS and OS have good recognition ability. In the training and validation set, the calibration curve shows that the predicted value of the nomograms for CSS and OS are highly consistent with the actual observed value **(** **Figure 3**). It shows that the nomograms have good accuracy. The AUC at 3-, 5-, and 8-years was 89.6,87.2, and 85.1, respectively, in the training set for CSS, and in the validation set for CSS, the AUC at 3-,5-, and 8-years was 89.9,88.4, and 85.7, respectively. In the training set for OS, the AUC at 3-, 5- and 8-years was 77.0,75.0, and 75.0, respectively, and in the validation set for OS, the AUC at 3-, 5- and 8-years was 77.4,75.4 and 74.5. The results show that the nomograms are very discriminative (**Figure 4**). The external validation set in time for predicting CSS was 0.903(95%CI:0.891-0.915), and the external validation set in time for predicting OS was 0.795(95%CI:0.785-0.805). The AUC at 1-and 2-year in the external validation set in time for CSS was 89.2 and 90.3, and the AUC in the external validation set in time for OS was 78.3 and 89.8 (**Figure S1**). Due to more than 75% of patients being classified as unknown GS groups, thus to bias the results, so we retrained the models after removing the unknown GS. The results showed that the C-index of the training set for CSS after removing the unknown GS is 0.785(95%CI:0.754-0.816), and the C-index of the training set for OS is 0.675(95%CI:0.657-0.693). The C-index of the validation set for CSS is 0.763(95%CI:0.708-0.818), and the C-index of the validation set for OS is 0.665(95%CI:0.640-0.690). Moreover, the AUC also showed that the model readiness and reliability would be decreased significantly after deleting the unknown GS (**Figure S2**).

### Clinical Application of the Nomograms

**Figure 5:**
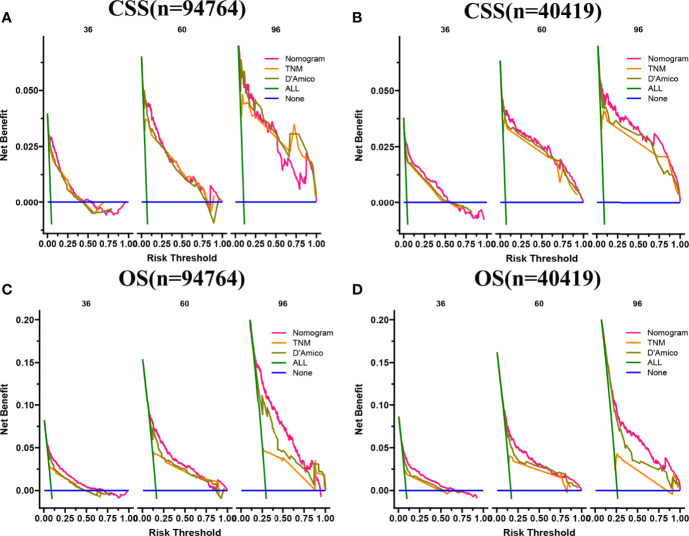
DCA of the nomograms for predicting CSS and OS. **(A)** The nomogram for CSS at 3,5-year showed the best clinical potential value in the training set, while the nomogram for CSS at 8-year had no apparent advantages over the other two. **(B)** In the validation set, the nomogram for CSS at the 3,5,8-year showed the best clinical potential value, followed by the D’Amico risk stratification system and TNM staging system. **(C, D)** The nomogram for OS at 3-,5-,8-year showed the best application potential in both the training and validation sets, followed by D’Amico risk stratification and TNM staging.

**Figure 6:**
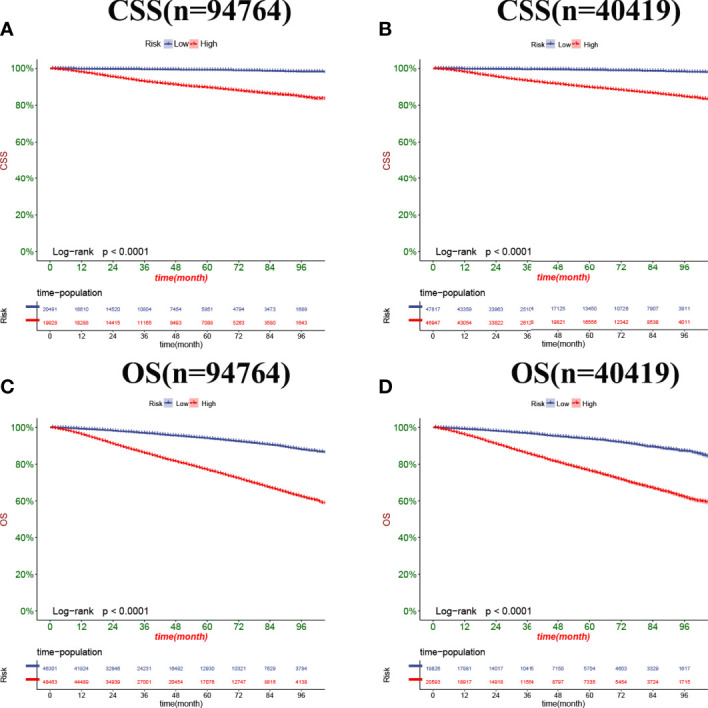
Kaplan-Meier curves of patients in the low-risk and high-risk groups. The K-M curve showed that the CSS rate of the patients in the high-risk group was significantly lower than that in the low-risk group both in the training set **(A)** and validation set **(B)**. The K-M curve showed that the OS rate of the patients in the high-risk group was significantly lower than that in the low-risk group both in the training set **(C)** and validation set **(D)**.

**Figure 7:**
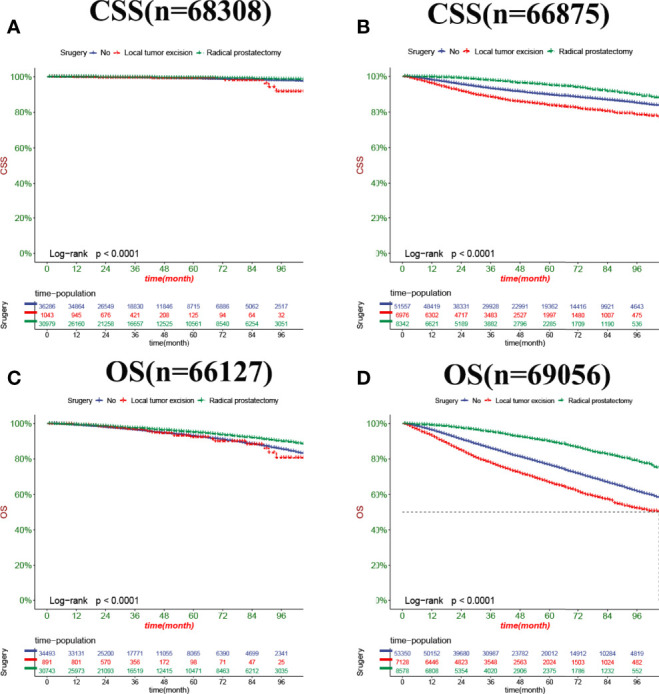
Kaplan-Meier curves of patients with a different surgery. **(A)** The CSS rate of patients in the low-risk group underwent different surgery. **(B)** The CSS rate of patients in the high-risk group underwent different surgery. **(C)** The OS rate of patients in the low-risk group underwent different surgery. **(D)** The OS rate of patients in the high-risk group underwent different surgery.

In both the training set and the validation set for CSS and OS, DCA suggested that the nomograms had good clinical potential value (**Figure 5**). The nomograms for CSS at the 3,5,8-year validation set showed the best clinical potential value, followed by the D’Amico risk stratification and TNM staging systems. The nomogram for CSS at 3,5-year also showed the best clinical potential value in the training set. In contrast, the nomogram for CSS at 8-year had no apparent advantages over the other two, indicating that the nomogram is close to the other two models in the long term and does not show apparent advantages. The nomogram for OS at 3,5,8 years showed the best application potential in both the training and validation set, followed by D’Amico risk stratification and TNM staging. Based on the nomogram, we calculated each patient’s risk value and the optimal cutoff value using the ROC curve. Patients were classified into the high-risk group (total score ≥293.59) and the low-risk group (total score <293.59) for predicting CSS, and patients were divided into the high-risk group (total score ≥184.88) and the low-risk group (total score <184.88) for predicting OS. The K-M curve showed that the CSS and OS rate of the patients in the high-risk group was significantly lower than that in the low-risk group both in the training and validation set (**Figure 6**). The 3-year, 5-year, and 8-year CSS rates of the patients in the high-risk group were 93.2%, 89.6%, and 84.7%, respectively. The low-risk group’s 3-year, 5-year, and 8-year CSS rates were 99.6%, 99.2%, and 98.2%, respectively. The 3-year, 5-year, and 8-year OS rates of the patients in the high-risk group were 86.0%.0,76.7%, and 62.2%, respectively. The low-risk group’s 3-year, 5-year, and 8-year OS rates were 96.8%, 93.9%, and 87.8%, respectively. We found that patients in the high-risk group had the highest CSS and OS rate for undergoing radical prostatectomy, but most patients did not receive surgery. Most patients in the low-risk group underwent radical prostatectomy or non-surgical treatment, with no significant difference in patient CSS and OS rate (**Figure 7**).

## Discussion

This study developed a nomogram using a population-based SEER database to predict CSS in elderly PC patients. With 135,183 patients included in this study, we successfully developed a nomogram to predict 3,5, and 8-year CSS in elderly PC patients, while internal validation demonstrated good calibration and discrimination of this nomogram. The nomogram consists of multiple independent prognostic factors, including age, marriage, Race, PSA, biopsy GS, radiotherapy, chemotherapy, surgical, tumor grade, and TNM stage.

In the past 170 years, PC has evolved from a rare disease to the most common non-cutaneous cancer; with the popularity of PSA screening, more and more patients can be detected in the early stages of the disease. PSA is the most common tumor marker in PC screening, although high levels of PSA in benign prostatic hyperplasia and prostatitis reduce PSA specificity as a cancer marker. However, many studies show that high levels of PSA are associated with a poor prognosis in prostate cancer patients (31). However, a previous study reported that low levels of PSA decreased the CSS in PC patients (32). Interestingly, different predictive models have opposite conclusions on the effects of PSA for the bone metastasis patients in PC, and the Indonesian nomogram suggests that higher PSA levels are associated with a worse prognosis (33). The SEER database also confirmed that high PSA levels were associated with poor prognosis (34). However, the Japanese nomogram suggests that PC patients with higher PSA suggest a better prognosis (35).In previous studies, PSA was generally divided into three grades: <4,4-10 and> 10 ng/ml (36, 37). This standard also graded our study. We also confirmed that PSA> 10 ng/ml was associated with a poor prognosis, consistent with most previous reports. However, the patient prognosis of PSA< 4 ng/ml and PSA 4-10ng/ml was inconsistent with the expression level of PSA. Considering the particularity of elderly PC patients, the PSA of most patients may be high, and the patients with PSA< 4 ng/ml are very few, which may bias the results. Secondly, it is reported that PSA 4-10 ng/ml itself is a “gray area” in PC (38), so the previous PSA classification criteria for elderly PC patients can not accurately respond to the actual situation. Moreover, we also made the corresponding nomogram for predicting OS, which showed the same conclusion that patients with PSA< 4 ng/ml had worse OS than PSA 4-10 ng/ml. Considering that it is inconsistent with clinical practice, we also recommend subsequent researchers adopt a new PSA classification standard for elderly PC patients over 65 years of age: PSA <10 ng/ml,10-20 ng/ml,> 20 ng/ml, instead of 4 ng/ml as the intermediate value, which may avoid outcome bias. SEER database-based studies have divided PSA into three levels: <10 ng/ml,10-20 ng/ml, and> 20 ng/ml. Most prediction models confirmed that PSA greater than 10 rather than 4 is associated with a poor prognosis (28, 34).

GS as an essential tool for predicting the prognosis of patients with PC has been revised multiple times since being proposed. The most common risk stratification for prostate cancer is the D’Amico classification, also used by the National Comprehensive Cancer Network (39), which divides Gleason scores into three Gleason score groups (2 – 6,7 and 8 – 10). However, the current GS system still has vast defects, especially with a total score of 7. The patient prognosis of GS 3 + 4 and GS 4 + 3 is very different, so the simple GS does not accurately predict the prognosis of PC patients. Therefore, a study developed a nomogram for patients with GS 4 + 3 and GS 3 + 4, which showed that patients with GS 4 + 3 had a worse prognosis than patients with GS 3 + 4 (28), and our study reached the same conclusion.Meanwhile, our nomogram showed that GS> 8 is associated with a worse prognosis, consistent with previous reports, but patients with GS<6 have a worse prognosis than those with GS7. We consider that the majority of patients did not undergo a needle biopsy for PC, resulting in more than 75% of cases being classified as biopsy GS unknown group, thus bias the results. After removing the unknown GS, the model was trained again. The result showed that deleting this part of the patients caused a significant decrease in the accuracy and reliability of the model and, therefore, poor availability.

PC treatment uses active monitoring, surgical resection, and androgen deprivation (ADT), combined with radiotherapy and chemotherapy. The surgical methods of PC mainly include: radical prostatectomy (RP) and local tumor resection (LTR), our prediction model showed that patients with RP had better outcomes than patients treated without surgery, which is consistent with previous reports (40, 41), and patients with LTR had the worst prognosis, considering many low-risk patients only need active monitoring rather than surgical treatment can obtain good prognosis, and many elderly PC patients who need RP but choose palliative surgery due to inability of tolerating prolonged general anesthesia, life expectancy is less than 10 years, etc. Therefore, our study found that for elderly patients with PC, patients with local tumor resection instead had a worse prognosis than those who did not receive surgical treatment. Radiation therapy (RT) is a conventional treatment method for PC patients, and it is mainly used clinically to treat patients with medium-risk or high-risk nonmetastatic prostate cancer (42, 43). RT is noninvasive and does not require considering the cardiorespiratory risks arising from systemic or local anesthesia. Therefore, it can be used to treat intolerable elderly patients with PC. At the same time, it does not require hospitalization, improving patient compliance while also reducing hospitalization costs. More importantly, RT can avoid some side effects of surgery, such as urinary incontinence. Our findings showed that elderly PC patients with RT had better outcomes than patients without RT, consistent with previous studies (44). Thus, RT is becoming a key component of multimodal therapy at multiple stages of PC. Unlike most solid tumors, chemotherapy (CT) is not the primary treatment for PC. Almost all PC will eventually develop metastatic castration-resistant prostate cancer (mCRPC), and in the 1970s and 1980s, although many chemotherapeutic drugs were tested in CRPC. Most drugs were tested in phase II clinical trials, and although many seemed promising, none were ultimately shown to prolong survival (45). As most of the patients did not receive chemotherapy, our predictive model also did not show a survival advantage of chemotherapy for elderly PC patients.

Cancer is now commonly evaluated through the tumor-lymph node-metastasis (TNM) system, which was previously considered the “gold standard” for staging and a benchmark for prognosis (46). Most nomograms of PC prognosis (28, 34) included traditional TNM stages, showing that T4 has the worst prognosis compared to other T stages, and patients with distant and lymph node metastasis had a worse prognosis compared to patients without distant and lymph node metastasis, which is consistent with our findings.

The social support provided by marital relationships can promote a healthy lifestyle and increase healthcare-seeking behavior, so marriage is associated with favorable outcomes for most cancer patients. Charlotte Salmon et al. emphasized the elevated risk of PC in single men (47). Libby Ellis et al. also demonstrated that marital status in prostate cancer patients is associated with prognosis (48). Our prediction model also shows the relationship between marital status and prognosis, proving that married patients often indicate a good prognosis, which may be related to the influence of marriage on mood, a medical decisions, etc. In addition, it may be due to the responsibility of married families. Married cancer patients can detect physical abnormalities and actively cooperate with treatment (49).In terms of race or ethnicity, previous studies showed that black men had the highest PC incidence and mortality rates (50). David A Siegel et al. reported 5-year survival (6) between 2001 and 2016, showing that 5-year survival was higher among other ethnic men (84.4%) and white men (82.8%) than among black men (79.1%). Our results showed that elderly Black and white patients with PC had a worse prognosis than other races, consistent with previous reports. Although survival rates of PC patients of different ages vary by stage, however, compared with younger patients, elderly patients likely secondary to the rapid development of resistant PC, reduced ability to receive available treatment, and the effects of comorbidities often have lower long-term survival (51), which is also supported by our results.

Although the nomogram based on the SEER database has good accuracy, there are potential limitations. First is the lack of critical clinicopathological variables, such as smoking, alcohol consumption, hemoglobin, etc. In addition, for PC patients, PSA is an important indicator related to prognosis. However, it was not included in the SEER database until 2010, so we can only choose the data after 2010 for building the prediction model. Meanwhile, ADT, as one of the non-surgical treatment options for PC patients, is usually used for high-risk local or systemic advanced disease that is not suitable for radical surgery. However, it lacks ADT-related data in the SEER database, so our model also lacks the relationship between ADT and prognosis.

Furthermore, database-based studies are all retrospective, which may risk selection bias. Further multi-center prospective studies with a large sample are needed to validate this nomogram. Finally, although our nomogram did not consider all related prognostic variables, we still included key variables and validated them, so there would not be a significant deviation.

## Conclusion

We explored the factors influencing CSS and OS in elderly patients with PC and found that age, race, marriage, PSA, biopsy GS, surgical approach, radiotherapy, chemotherapy, tumor grade, and TNM stage were independent risk factors affecting patients’ CSS and OS. We established nomograms to predict the CSS and OS in elderly patients with PC. The models have been internally validated with good accuracy and reliability, and they can help make better clinical decisions for clinicians and patients.

## Data Availability Statement

The original contributions presented in the study are included in the article/**Supplementary Material**. Further inquiries can be directed to the corresponding author.

## Author Contributions

ZZ and CZ designed the study. ZZ, JW, JL, MJL, LJ and MXL collected and analyzed the data. ZZ drafted the initial manuscript. CZ, TM, JL, XT and DH revised the article critically. XW, XT, CZ, JL, DH, MJL reviewed and edited the article. All authors approved the final manuscript. All authors contributed to the article and approved the submitted version.

## Funding

Special Key Project of Chongqing Technology Innovation and Application Development (No. Cstc2019jscx-tjsbX0003), Yunnan Education Department of Science Research Fund (No. 2020 J0228), Kunming City Health Science and Technology Talent “1000” training Project (No. 2020- SW (Reserve)-112), Kunming Health and Health Commission Health Research Project (No. 2020-0201-001), and Kunming Medical Joint Project of Yunnan Science and Technology Department (No. 202001 AY070001-271).

## Conflict of Interest

The authors declare that the research was conducted in the absence of any commercial or financial relationships that could be construed as a potential conflict of interest.

## Publisher’s Note

All claims expressed in this article are solely those of the authors and do not necessarily represent those of their affiliated organizations, or those of the publisher, the editors and the reviewers. Any product that may be evaluated in this article, or claim that may be made by its manufacturer, is not guaranteed or endorsed by the publisher.

## Supplementary Material

Supplementary Figure 1 The AUC at 1-,2-year in the external validation set in time. A: The AUC at 1-,2-year in the external validation set for CSS was 89.2 and 90.3. B: The AUC in the external validation set for OS was 78.3 and 89.8.

Supplementary Figure 2 The AUC at 3-,5-,8-year for predicting CSS and OS after deleting the unknown GS. A: The AUC at 3-,5-,8-year for predicting CSS after deleting the unknown GS was 76.4,82.6, and 82.3.B: The AUC at 3-,5-,8-year for predicting OS after deleting the unknown GS was 65.9,77.9 and 73.8.
