# Supplementary figures and images for: Development and Validation of Nomograms to Predict Cancer-Specific Survival and Overall Survival in Elderly Patients With Prostate Cancer: A Population-Based Study
Source: Front Oncol. 2022 Jun 23;12:918780. doi: 10.3389/fonc.2022.918780 (PMC9259789; doi:10.3389/fonc.2022.918780)

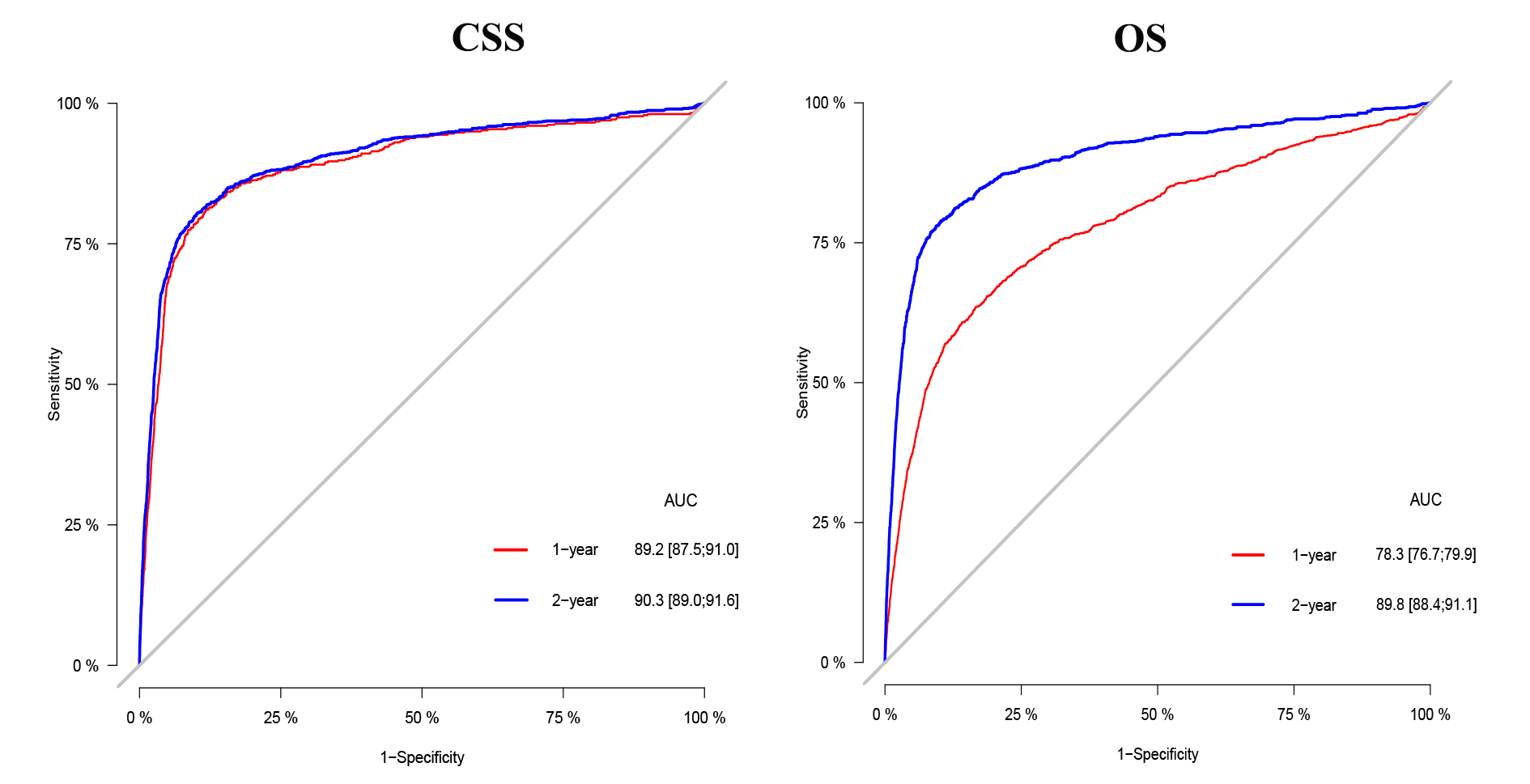

Supplement: Supplementary Figure 1 — The AUC at 1-,2-year in the external validation set in time. A: The AUC at 1-,2-year in the external validation set for CSS was 89.2 and 90.3. B: The AUC in the external validation set for OS was 78.3 and 89.8. [file Image_1.tif]

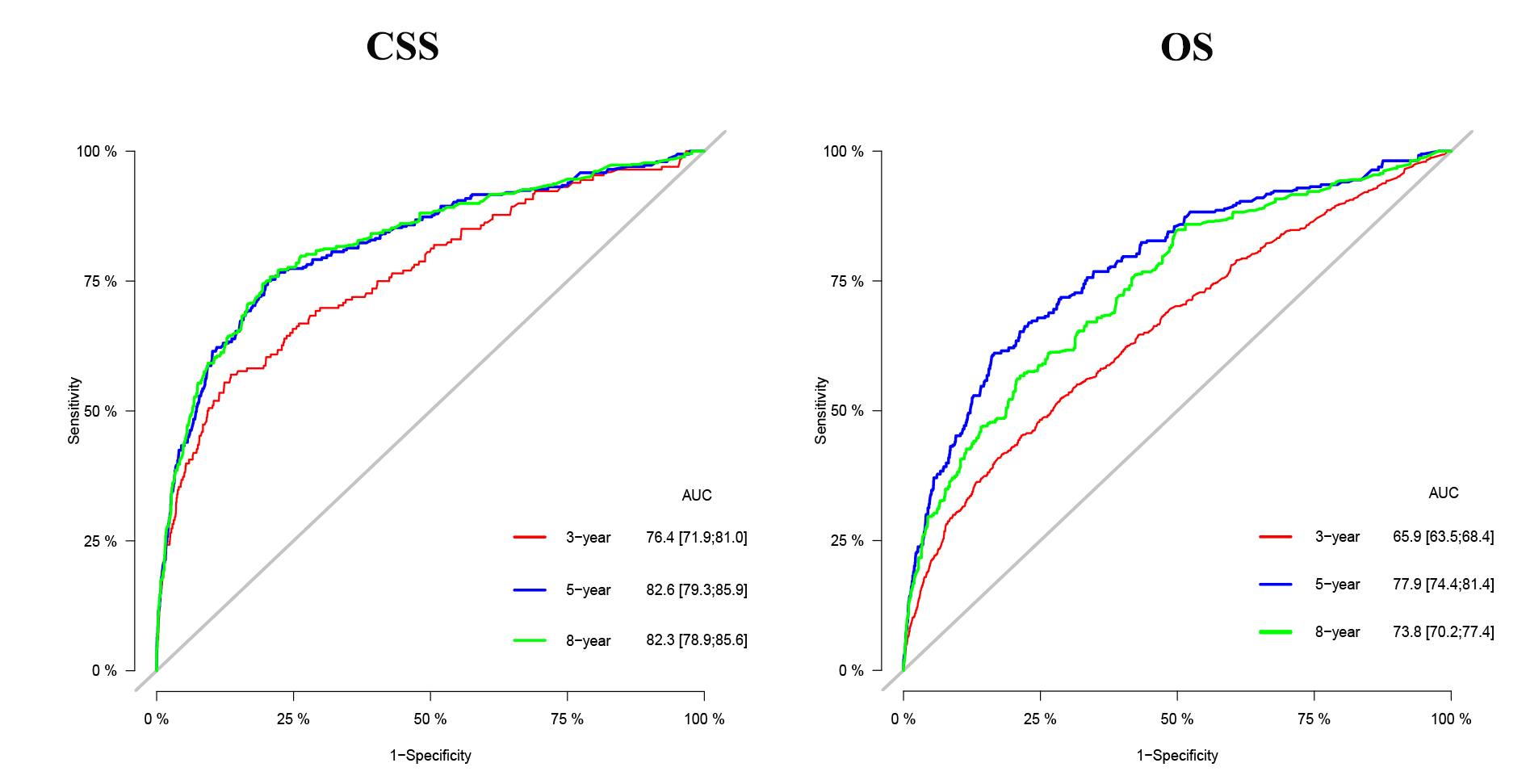

Supplement: Supplementary Figure 2 — The AUC at 3-,5-,8-year for predicting CSS and OS after deleting the unknown GS. A: The AUC at 3-,5-,8-year for predicting CSS after deleting the unknown GS was 76.4,82.6, and 82.3.B: The AUC at 3-,5-,8-year for predicting OS after deleting the unknown GS was 65.9,77.9 and 73.8. [file Image_2.tif]
